# Estimating the Cost-Effectiveness of One-Time Screening and Treatment for Hepatitis C in Korea

**DOI:** 10.1371/journal.pone.0167770

**Published:** 2017-01-06

**Authors:** Do Young Kim, Kwang-Hyub Han, Byungyool Jun, Tae Hyun Kim, Sohee Park, Thomas Ward, Samantha Webster, Phil McEwan

**Affiliations:** 1 Department of Internal Medicine, Yonsei University College of Medicine, Seoul, South Korea; 2 Department of Preventive Medicine, Cha University College of Medicine, Kyung-Gi Province, South Korea; 3 Graduate School of Public Health, Yonsei University, Seoul, South Korea; 4 HEOR Ltd, Singleton Court Business Park, Monmouth, Wales, United Kingdom; 5 School of Human & Health Sciences, Swansea University, Wales, United Kingdom; Chang Gung Memorial Hospital Kaohsiung Branch, TAIWAN

## Abstract

**Background and Aims:**

This study aims to investigate the cost-effectiveness of a one-time hepatitis C virus (HCV) screening and treatment program in South Korea where hepatitis B virus (HBV) prevails, in people aged 40–70, compared to current practice (no screening).

**Methods:**

A published Markov model was used in conjunction with a screening and treatment decision tree to model patient cohorts, aged 40–49, 50–59 and 60–69 years, distributed across chronic hepatitis C (CHC) and compensated cirrhosis (CC) health states (82.5% and 17.5%, respectively). Based on a published seroepidemiology study, HCV prevalence was estimated at 0.60%, 0.80% and 1.53%, respectively. An estimated 71.7% of the population was screened. Post-diagnosis, 39.4% of patients were treated with a newly available all-oral direct-acting antiviral (DAA) regimen over 5 years. Published rates of sustained virologic response, disease management costs, transition rates and utilities were utilised.

**Results:**

Screening resulted in the identification of 43,635 previously undiagnosed patients across all cohorts. One-time HCV screening and treatment was estimated to be cost-effective across all cohorts; predicted incremental cost-effectiveness ratios ranged from $5,714 to $8,889 per quality-adjusted life year gained. Incremental costs associated with screening, treatment and disease management ranged from $156.47 to $181.85 million USD; lifetime costs-offsets associated with the avoidance of end stage liver disease complications ranged from $51.47 to $57.48 million USD.

**Conclusions:**

One-time HCV screening and treatment in South Korean people aged 40–70 is likely to be highly cost-effective compared to the current practice of no screening.

## Introduction

The hepatitis C virus (HCV) is a leading cause of life-threatening liver disease and a major global public health issue. In South Korea, the prevalence of chronic hepatitis C (CHC) is estimated at 0·78%, with the majority of patients aged over 40 years [1]. Progression of CHC to end-stage liver disease (ESLD), including compensated cirrhosis (CC), liver failure (decompensated cirrhosis [DC]) and hepatocellular carcinoma (HCC), sequelae that often require transplant, is associated with significant mortality and imposes a significant financial burden upon healthcare systems [1,2]. The goal of hepatitis C treatment is to eradicate the HCV infection; this is assessed via the achievement of a sustained virologic response (SVR), defined as undetectable serum HCV RNA at the end of treatment [3]. Rates of SVR can vary significantly between treatments, depending on factors such as HCV genotype and severity of disease [3]. Further, a large proportion of countries observe relatively low rates of treatment uptake, influenced by poor treatment tolerability and low rates of patient identification due to the asymptomatic nature of the disease in its early stages [4]. In recent years, the therapy landscape for HCV has expanded significantly with the introduction of all-oral direct-acting antiviral (DAA) regimens. These are associated with high efficacy and improved tolerability relative to historical standard of care (pegylated interferon-alpha combined with ribavirin [PEG-IFNα+RBV]).

From a public health perspective, efforts are currently focused toward the implementation of treatment strategies that may decrease rates of onward transmission [5]. Globally, there has been considerable interest in assessing the cost-effectiveness of screening programs for HCV; analyses have been performed in several countries with a high prevalence of HCV including the US, Japan and Egypt [6–12]. However, due to the differing epidemiological characteristics associated with the HCV population in South Korea [3], and since healthcare systems differ significantly between countries, generalizing results from one country to another is not appropriate.

Despite the implementation of a screening program for hepatitis B virus (HBV) in South Korea, there is not currently a screening program for HCV, and the economic and clinical outcomes associated with such a strategy have not been evaluated. Therefore, the objective of this study was to investigate the cost-effectiveness of implementing a screening and treatment program in South Korea.

## Methods

Cost-effectiveness was assessed through the accumulation of lifetime costs, life years (LYs) and quality-adjusted life years (QALYs) associated with certain screening and treatment scenarios modeled.

The most common HCV genotypes in South Korea are genotype 1b (45–59%) and 2a (26–51%); types 1a, 2b, 3, 4, and 6 are rare [5,13]. As the predominating subtypes of HCV in South Korea (>95%), only genotypes 1 (G1) and 2 (G2) infection were considered [5,13]. The cost-effectiveness of screening and treatment was investigated in patients aged 40–69 years, due to their predicted dominance of the prevalent HCV population in South Korea [14]. The analyses were stratified into three cohorts according to their estimated age at screening: i) 40–49 years; ii) 50–59 years; and iii) 60–69 years.

### Screening

Overall HCV prevalence was estimated from published South Korean population statistics, and the number of patients currently aware of infection was estimated from Health Insurance Review and Assessment (HIRA) claims data [15,16]. Published Korea-specific data were used to inform the age-dependent prevalence of infection, resulting in estimates of 0·60%, 0·80%, and 1·53% in those aged 40–49 years, 50–59 years, and 60–69 years, respectively [1]. It was estimated that approximately 71·7% of the population would undergo screening for HCV during a one-time medical check-up, based upon the National Screening Program for Transitional Ages [17].

It was assumed that all patients in each cohort were screened simultaneously at model initiation. The successful identification of HCV positive subjects in South Korea is confirmed through a series of tests, described based upon expert clinical opinion: HCV antibody test, HCV RNA quantitative test and ultrasound. A false positive rate of 53·5% for the initial HCV antibody test was incorporated, but it was assumed that the subsequent HCV RNA test provided a definitive diagnosis [18].

### Treatment

Upon diagnosis of HCV, an estimated 39.4% of patients were allocated treatment over a five-year period [19]. As no Korean-specific data relating to the timing of treatment initiation after HCV diagnosis was available, this was informed by expert opinion: it was assumed that 60% of eligible patients were treated in the first year, with the remainder treated in equal proportions over the subsequent four years. Those not treated in the first year may progress to more advanced stages of liver disease or death, thus becoming ineligible for treatment.

Under base case assumptions, allocated patients received treatment with newly available DAAs, in line with current reimbursement guideline available in South Korea. The distribution of treated patients across treatments is presented in Table 1, with treatments assigned based on genotype, prevalence of resistance-associated variants (RAV) at baseline, and health state (chronic hepatitis C or compensated cirrhosis). Under base case settings, in the absence of market share data, and where multiple therapies are available to a patient subgroup, an equal market share was assumed. Efficacy data was sourced from appropriate clinical trials (Table 1) [20–25]. Treatment-related adverse events, disutility, and discontinuation, were not considered in this analysis.

**Table 1:** Treatment parameters.

Genotype	Sub-genotype	RAV status	Health state	Treatment	Treatment duration (weeks)	Total drug cost ($USD)	Monitoring cost ($USD)	SVR
Genotype 1 (52.7%) [5]	Non-genotype 1b (14.1%) [5]	N/A	Chronic hepatitis C (82.5%)	DCV/SOF	12	22,575	641	100.0% [20]
				LDV/SOF	12	22,559	641	100.0% [21]
			Compensated cirrhosis (17.5%)	DCV/SOF	12	22,575	641	91.0% [22]
				LDV/SOF/RBV	12	22,585	641	100.0% [21]
	Genotype 1b (85.9%) [5]	RAV–ve (86.2%) [22]	Chronic hepatitis C (82.5%)	DCV/ASV	24	7,733	926	96.0% [23]
			Compensated cirrhosis (17.5%)					
		RAV +ve (13.8%) [22]	Chronic hepatitis C (82.5%)	DCV/SOF	12	22,575	777	100.0% [20]
				LDV/SOF	12	22,559	777	100.0% [21]
			Compensated cirrhosis (17.5%)	DCV/SOF	12	22,575	777	91.0% [22]
				LDV/SOF/RBV	12	22,585	777	100.0% [21]
Genotype 2 (47.3%) [5]	N/A	N/A	Chronic hepatitis C (82.5%)	SOF/RBV	12	19,515	641	97.0% [24]
	N/A	N/A	Compensated cirrhosis (17.5%)	SOF/RBV	16	26,021	691	100.0% [25]

ASV, asunaprevir; DCV, daclatasvir; LDV, ledipasvir; N/A, not applicable; RAV, resistant-associated variant; RBV, ribavirin; SOF, sofosbuvir; SVR, sustained virologic response; USD, United States Dollar.

### Model

**Fig 1:**
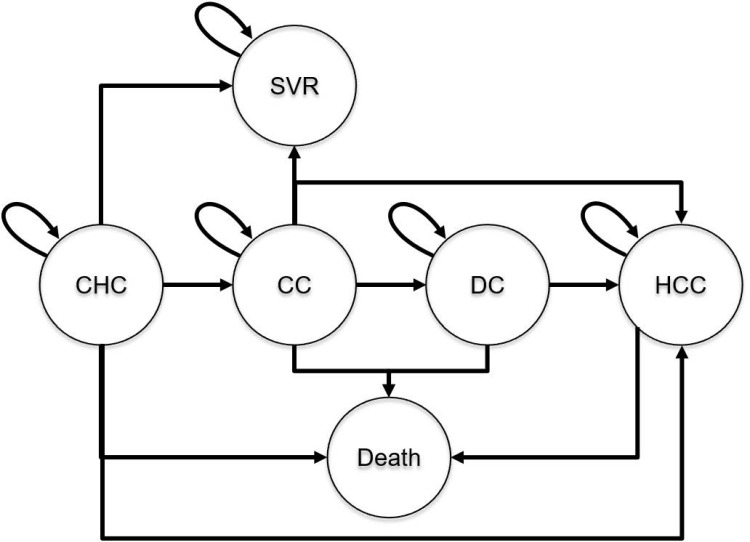
Markov model flow diagram.

The model utilized for the analysis was a previously published and validated CHC natural history Markov model, with an incorporated screening and treatment decision tree [26–36]. This was adapted to the South Korean setting. The natural history model is designed to progress a cohort of patients in annual cycles through hepatitis C health states, which include CHC, CC, DC, HCC, and death. The model flow diagram is presented in Fig 1. Annual health state transition rates are reported in Table 2. All-cause mortality rates are estimated through the incorporation of South Korean-specific life tables [37].

**Table 2:** Health state transition rates.

	Mean	SE	Source
**CHC and CC stages**			
CHC -> CC	0.065	0.011	Nakamura 2008 [12]
CHC -> HCC	0.016	0.004	Nakamura 2008 [12]
**Complication stages**			
CC -> DC	0.021	0.006	Imazeki 2005 [38]
CC -> HCC	0.043	0.008	Hayashida 2002 [39]
DC--> HCC	0.083	0.022	Nakamura 2008 [12]
DC--> Death (1st year)	0.153	0.022	Nakamura 2008 [12]
HCC--> Death (2nd year+)	0.200	0.012	Nakamura 2008 [12]

CC, compensated cirrhosis; CHC, chronic hepatitis C; DC, decompensated cirrhosis; HCC, hepatocellular carcinoma; HCV, hepatitis C virus; SE, standard error.

Patients enter the model immediately after screening and are initially distributed across CHC (82·5%) and CC (17·5%) health states [5]. According to published data, it was assumed that 52·7% of patients had HCV G1 infection, and the remainder had G2 [5].

The model considers three patient populations:

1. Undiagnosed subjects: This captures subjects that are not identified through screening who have no prior knowledge of infection, thus are assumed to experience uninterrupted disease progression. Due to their not being identified through screening, they are assumed not to incur the costs associated with the management of CHC and CC, and only incur costs when they present to the healthcare system at the ESLD stages of DC and/or HCC.
2. Subjects diagnosed but not treated: These subjects are identified through screening, but are not allocated treatment, thus also experience uninterrupted disease progression. They incur all health state management costs.
3. Subjects diagnosed and treated: These subjects are identified through screening and are allocated treatment over the first and subsequent four years of the simulation. Subjects are stratified by those that achieve SVR and those that do not, according to treatment efficacy rates. Subjects that achieve SVR experience no further disease progression and thus no further health state management costs. Those that do not achieve SVR resume disease progression and may incur health state management costs. All subjects incur the full costs associated with HCV treatment.

A fourth patient population exists: subjects that have previously been diagnosed and are currently aware of their infection status; however, as this population will have no impact on the predicted cost-effectiveness of future screening, due to their incorporation in both arms of the analysis, they are not considered in this study.

#### Cost and health utility values

The analysis takes a healthcare system perspective and considers only direct medical costs, inflated to 2016 values [40]. Indirect costs, such as those associated with absenteeism and presenteeism, were not considered in the analysis. All costs incorporated in the analysis were converted from South Korean Won (KRW) to United States Dollars (USD) at a conversion rate of $1:₩1,108.21 [41].

The costs for the HCV antibody test, HCV RNA quantitative test and ultrasound were $3·49, $147.33 and $61·43, respectively [42].

Within the natural history model, costs are applied to patients annually based on their health state; the costs of treatment and monitoring are applied during the year of treatment only, as a per event cost. The costs for drugs and monitoring are provided in Table A and Table B in S1 File, respectively [42,43].

The total costs of treatment were sourced from Korean list prices, and monitoring costs stratified by treatment were estimated based on HIRA claims data (Table 1) [42,44]. Health state costs were taken from published literature specific to South Korea; whereas, health state utility values were taken from Japanese literature in the absence of South Korean data (Table 3). Costs and utilities were discounted annually at a rate of 5%.

**Table 3:** Health state costs and utility estimates.

	Cost ($USD)	Source	Utility	Source
**CHC and CC stages**				
CHC	972.73	Kim 2016 [45]	0.92	Ishida 2012 [46]
CC	1,238.02	Kim 2016 [45]	0.86	Okita 2007 [47]
**Complication stages**				
DC	6,468.01	Kim 2016 [45]	0.67	Okita 2007 [47]
HCC	6,366.94	Kim 2016 [45]	0.38	Nakamura 2008 [12]
**SVR**				
From CHC and CC	0	Assumed	0.96	Ishida 2012 [46]

CC, compensated cirrhosis; CHC, chronic hepatitis C; DC, decompensated cirrhosis; HCC, hepatocellular carcinoma; SVR, sustained virologic response

### Analyses

The analyses undertaken focus on three key elements relevant to the cost-effectiveness of a screening and treatment program in South Korea:

1. *The cost-effectiveness of screening and treating*, *and the impact on complication event incidence and related cost-offsets*: Screening and treatment was compared to a scenario of no screening, in which no additional cases of HCV were identified over the modelled time horizon, for the three patient cohorts. Treatment with a DAA regimen was initiated in a proportion of patients, post-screening. Resultant event incidence and cost-offset results were compared to generate cost-effectiveness estimates. Additional analyses, focusing on individual subgroups (defined by genotype and RAV status) were performed to assess the implications of utilizing individual treatment regimens.
2. *The relationship between HCV prevalence*, *treatment uptake and cost-effectiveness*: The cost-effectiveness of screening and treatment is dependent upon the prevalence of HCV amongst those screened, as well as the uptake rate of treatment once patients have been identified [25]. As such, cost-effectiveness in the three patient cohorts was estimated as a function of HCV prevalence and treatment uptake.
3. *The impact of timing of treatment upon events avoided and cost-offsets*: Published data for US HCV patients have demonstrated that, once diagnosed, treating patients as early as possible results in reduced lifetime disease management cost and increased QALYs [25]. Therefore, in order to quantify the relationship between the timing of treatment and cost-effectiveness in the South Korean population, the period over which treatment was allocated was shortened to one and three years. Consistent with the base case analysis, within the three-year scenario, 60% of patients initiated therapy in the first year, with the remainder allocated therapy in equal proportions over the remaining two years, respectively. Under the one-year scenario, all patients were treated in the first year post-diagnosis.

## Results

**Fig 2:**
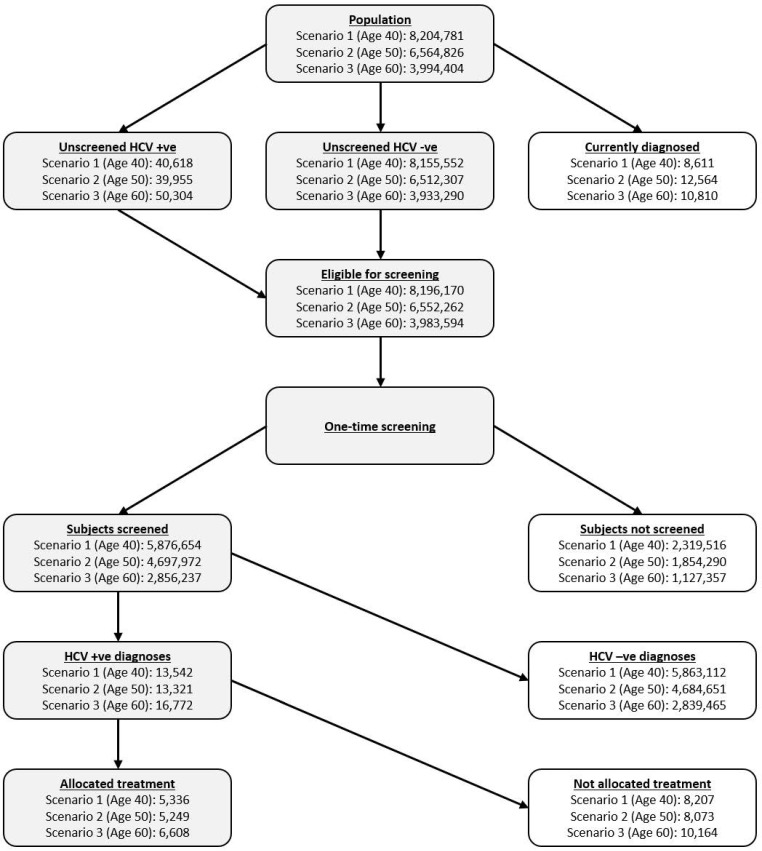
Screening flow diagram showing the derivation of the number of patients screened and allocated treatment across all patient populations.

Prevalence estimates and the corresponding derivation of numbers screened, diagnosed and treated are presented in Fig 2. The model predicted that a total of 17,193 individuals would be scheduled for treatment after screening had occurred (31·0% aged 40–49 years, 30·5% aged 50–59 years, and 38·4% aged 60–69 years). Across each age cohort, the introduction of a screening program was associated with additional cost, life years and QALYs (Table 4). The additional cost was a combination of HCV management and treatment costs. Resultant incremental cost-effectiveness ratios (ICERs) demonstrated that screening and treatment is expected to be highly cost-effective across patients aged 40–69, based upon a willingness-to-pay (WTP) threshold (GDP per capita 2015) of $27,512 [48]. Subgroup analyses demonstrated similar results, with ICERs ranging from $4,445–6,830, $5,267–8,237 and $6,661–10,868 in the 40–49 year, 50–59 year and 60–69 year cohorts, respectively (Table 5). Treatment with the combination of daclatasvir and asunaprevir (DCV+ASV) in RAV-negative genotype 1 patients resulted in the greatest cost-effectiveness, across all age groups.

**Table 4:** Base case cost-effectiveness results.

	Scenario 1 (Age 40–49)		Scenario 2 (Age 50–59)		Scenario 3 (Age 60–69)	
	Screening	No screening	Screening	No screening	Screening	No screening
**Absolute results**						
Total costs ($USD million)	379.05	214.33	355.77	199.30	406.08	224.23
Total life years	273,007	251,278	252,690	236,576	285,467	272,780
Total QALYs	238,195	209,365	220,262	197,397	248,686	228,229
**Incremental results**						
Costs ($USD million)	-	164.72	-	156.47	-	181.85
Life years	-	21,728	-	16,115	-	12,687
QALYs	-	28,830	-	22,865	-	20,457
**Cost-effectiveness ($USD)**						
ICER ($/life year)	-	7,581	-	9,710	-	14,334
ICER ($/QALY)	-	5,714	-	6,843	-	8,889
**Cost-effectiveness (₩KRW)**						
ICER (₩/life year)	-	8,401,081	-	10,760,670	-	15,885,121
ICER (₩/QALY)	-	6,331,798	-	7,583,830	-	9,851,429

ICER, incremental cost-effectiveness ratio; QALY, quality-adjusted life year; USD, United States Dollar.

**Table 5:** Subgroup cost-effectiveness results.

Genotype	Subgroup	ICER ($USD)		
		Scenario 1 (Age 40–49)	Scenario 2 (age 50–59)	Scenario 3 (Age 60–69)
All genotypes	All patients (base case)	5,714	6,843	8,889
	All patients (100% LDV + SOF ± RBV market share*)	5,697	6,823	8,863
	All patients (100% DCV + SOF market share†)	5,730	6,863	8,916
Genotype 1	All patients (100% LDV + SOF ± RBV market share*)	5,010	5,971	7,664
	All patients (100% DCV + SOF market share†)	5,068	6,042	7,758
Genotype 1b	RAV +ve patients (100% LDV + SOF ± RBV market share)	6,568	7,915	10,433
	RAV +ve patients (100% DCV + SOF market share)	6,830	8,237	10,868
	RAV -ve (100% DCV + ASV market share)	4,445	5,267	6,661
Genotype non-1b	All patients (100% LDV + SOF ± RBV market share)	6,545	7,887	10,393
	All patients (100% DCV + SOF market share)	6,807	8,208	10,827
Genotype 2	All patients (100% SOF/R market share)	6,457	7,764	10,186

* 100% LDV + SOF ± RBV market share for genotype 1b RAV+ and non-genotype 1b

† 100% DCV+SOF market share for genotype 1b RAV+ and non-genotype 1b; DCV, daclatasvir; ICER, incremental cost-effectiveness ratio; LDV, ledipasvir; RAV, resistance-associated variant; RBV, ribavirin; SOF, sofosbuvir; USD, United State Dollar

**Fig 3:**
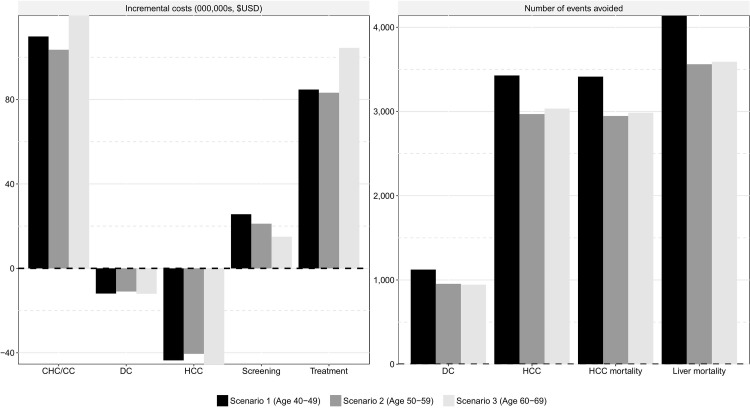
The number of events avoided and the incremental costs associated with a screening and treatment program.

The introduction of a screening and treatment program was estimated to reduce the total number of ESLD and mortality events considerably, with the largest reductions observed in those aged 40–49 years (Fig 3). The number of DC events avoided ranged from 944 to 1,123, whilst the number of HCC events avoided ranged from 2,969 to 3,427. Consequently, whilst there were additional costs associated with screening and treating patients, as well as the management of newly identified patients in the CHC and CC health states, costs associated with DC and HCC management were reduced.

**Fig 4:**
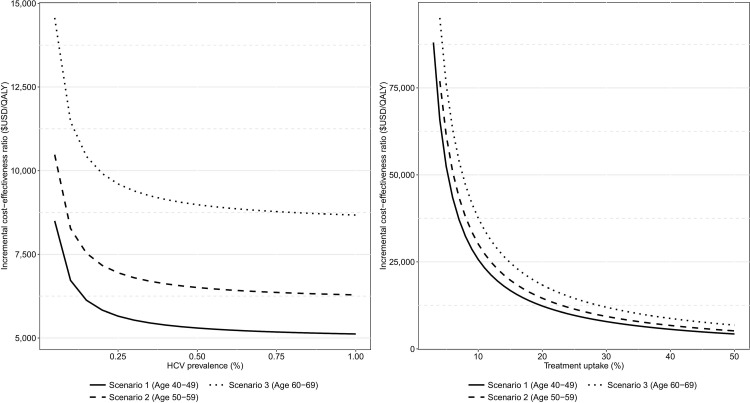
The relationship between the cost-effectiveness of screening and treatment, the prevalence of HCV amongst the general population and the rate of therapy uptake post diagnosis.

Fig 4 quantifies the relationship between HCV prevalence, treatment uptake and the cost-effectiveness of the screening and treatment program. Screening and treatment remained cost-effective at a $27,512/QALY threshold across all scenarios in which HCV prevalence was at least 0.04%. For the analysis in which baseline HCV prevalence was utilized, screening remained cost-effective when treatment uptake was at least 11%, 12% and 15% in the 40–49 year, 50–59 year and 60–69 year cohorts, respectively.

The relationship between the timing of treatment after diagnosis and its incremental costs and QALY gains is presented in Table 6. Across all scenarios, treating patients’ sooner after diagnosis was associated with reduced total cost and increased QALY gains compared to base case analyses in which patients were treated over a five-year time horizon. Decreasing the time period over which treatment was initiated increased cost-effectiveness estimates across all age groups.

**Table 6:** The relationship between the timing of treatment post-diagnosis and the cost-effectiveness of a screening and treatment program.

	Incremental results (versus no screening and treatment)		
	Costs ($USD, million)	QALYs	ICER ($USD/QALY)
**Scenario 1 (Age 40–49)**			
Base case	164.72	28,830	5,714
Treatment initiated over 3 years post-diagnosis	163.91	29,641	5,530
Treatment initiated in first year post-diagnosis	162.70	30,875	5,270
**Scenario 2 (Age 50–59)**			
Base case	156.47	22,865	6,843
Treatment initiated over 3 years post-diagnosis	155.73	23,577	6,605
Treatment initiated in first year post-diagnosis	154.63	24,674	6,267
**Scenario 3 (Age 60–69)**			
Base case	181.85	20,457	8,889
Treatment initiated over 3 years post-diagnosis	181.06	21,202	8,540
Treatment initiated in first year post-diagnosis	179.87	22,375	8,039

ICER, incremental cost-effectiveness ratio; QALY, quality-adjusted life year; USD, United States Dollar.

## Discussion

Results herein demonstrate that screening for HCV infection in South Korea is likely to be cost-effective for people aged between 40 and 69 years, when compared to no screening. Whilst this analysis has demonstrated cost-effectiveness results consistent with previous studies, the implementation of a screening program raises a number of important issues. For example, the costs associated with managing CHC and CC are considerable. This reflects both the large number of people newly diagnosed with HCV and the relatively high costs associated with managing patients with CHC infection in South Korea. For comparison, in the US, the cost of managing CHC ($209) is approximately 0.8% of the cost of managing DC ($27,845) [25]; in South Korea, the cost of managing CHC ($973) is approximately 15.0% of the cost of managing DC ($6,468). Several factors, such as difference in medical system, might be involved in difference in the ratio of managing cost for CHC and DC between US and Korea. But this comparison should be interpreted with caution because in Korea study, costs such as lab test, radiologic tests, biopsies, adverse events resulting from the management of CHC and its complications, and antiviral treatment cost for patients who were treated were all included. And especially for CHC state, the treatment uptake rate was high compared to other disease state which in result increased total CHC state cost. [6] In comparison, US study for CHC state only included one office visit, one CBC, one liver profile, and 1 HCV RNA test each year costs. [25]

Anyhow, despite the additional disease management cost incurred as a result of identifying a large number of previously undiagnosed HCV patients, a screening and treatment strategy remains highly cost-effective. Cost-effectiveness in this case is driven by the successful treatment of patients shortly after HCV diagnosis. This is because these individuals would otherwise not have been diagnosed until they presented with end-stage liver disease complications, at which point successful treatment is far less likely, leading to reductions in quality of life and significantly increased medical expenditure.

Increased rates of treatment uptake are shown to be associated with an improved cost-effectiveness profile. The advent and availability of novel DAAs that are well tolerated and associated with high rates of treatment success, in even in the most difficult to treat patients, presents a significant opportunity for the treatment of HCV, and are thus likely to result in greater treatment uptake rates than those currently observed.

The most significant finding in this study is that the cost-effectiveness of anti-HCV testing was the highest in subjects aged 40s. Actually, determination of the time point of HCV screening might be important to make a decision on national program and to implement a public health strategy. As seen in Tables 4 and 5, the overall and subgroup analysis for screening and treatment of HCV infection were cost-effective in all three scenarios. The reason why scenario 1 (age 40–49) is the most cost-effective could be explained by the fact that identification of relatively young patients and treatment initiation result in lowering disease progression and ultimately reducing overall costs related to the management of advance liver disease.

This analysis demonstrates that immediate treatment initiation is likely to be more cost-effective than delaying treatment post-diagnosis; such results are consistent with a previously undertaken screening analysis, based on a US birth cohort population. Further, recent analysis undertaken in a South Korean population, illustrated with the recently approved DAA regimen, DCV+ASV, concluded that treating patients immediately, rather than delaying treatment by 1,3,5,7, and 10 years, has significant benefit in terms of avoiding ESLD complications, improving quality of life and reducing cost [49].

There are relatively few studies that aim to quantify the prevalence of HCV in South Korea, much less estimate the distribution of prevalence rates across localized regions. However, several studies suggest that the prevalence of HCV in South Korea is likely to be endemic in particular regions [4,50]. Therefore, the results of this analysis should be considered within the broader context of the South Korean demographic. The feasibility of treating large numbers of patients across South Korea and the potential for geographical clustering have not been accounted for. As demonstrated herein, HCV prevalence can impact the cost-effectiveness of screening; in geographical areas with low prevalence, a large number of people will incur screening costs with limited opportunity to generate sufficient QALY gains from successfully treating patients. Consequently, whilst the analysis is valid across the South Korean population in general, the results presented in this analysis may overestimate cost-effectiveness in some regions and underestimate in others.

The potential requirement to re-treat patients failing to achieve SVR was not taken into consideration for this study. While modelling the health economic consequences associated with treatment failure in terms of disease progression has been included, modelling re-treatment is not straightforward. Uncertainty surrounding the timing of re-treatment combined with uncertainty as to the future standard of care when re-treatment is undertaken presents a number of challenges. The relevance and impact of this limitation will be influenced by the choice of initial therapy. However, as SVR rates continue to improve with the advent of DAA regimens, the requirement to re-treat will diminish.

There exists uncertainty in population level data, such as the prevalence of HCV infection in South Korea and the percentage of the total population screened. The analysis explored the impact of HCV prevalence on cost-effectiveness; however, the percentage screened was not varied. Based on the relatively low screening cost, the impact of increasing the percentage screened would improve the cost-effectiveness profile (assuming the same proportion are treated); this is because the cost savings and health benefits associated with successfully treating patients dominates the results. Furthermore, although it has not been possible to quantify the effect due to a lack of appropriate data, it is likely that the exclusion of societal costs within this modelling exercise results in an underestimation of cost-effectiveness. Previously published studies demonstrate that patients with hepatitis C experience significantly increased rates of absenteeism and presenteeism, compared to the general population [51–53].

In conclusion, this analysis has demonstrated that one-time screening for HCV in South Korea is likely to be highly cost-effective in people aged 40–69 years at current levels of treatment uptake. In support of this, results were relatively insensitive to modest changes in the rate of treatment uptake and the prevalence of HCV infection. With the recent availability of new DAA regimens such as DCV+ASV in Korea, the historical focus on treatment efficacy should perhaps be shifted to a focus on identification of undiagnosed patients. Results suggest that a national screening program in South Korea could significantly reduce the incidence of HCV-related ESLD complications and mortality, and offer an important initial step towards a national health policy aimed at managing HCV in the South Korean population.

## Supporting Information

S1 File Table A. Drug costs; Table B. Monitoring costs.
(DOCX)
