# Supplementary material for: Estimating the Cost-Effectiveness of One-Time Screening and Treatment for Hepatitis C in Korea
Source: PLoS One. 2017 Jan 6;12(1):e0167770. doi: 10.1371/journal.pone.0167770 (PMC5218507; doi:10.1371/journal.pone.0167770)
Supplement: S1 File — Table A. Drug costs; Table B. Monitoring costs. (DOCX) [file pone.0167770.s001.docx]

**Table A. Drug costs**

|  | **Cost ($USD)** | **Source** |
| --- | --- | --- |
| **Drug costs (weekly)** |  |  |
| DCV | 257.10 | Korean List price [42] |
| ASV | 65.11 | Korean List price [42] |
| SOF | 1624.12 | Korean List price [42] |
| LDV/SOF | 268.56 | Korean List price [42] |
| RBV | 2.17 | Korean List price [42] |
| **Total drug cost for genotype 1** |  |  |
| DCV/ASV | 7,733.05 | Korean List price [42] |
| DCV/SOF | 22,574.59 | Korean List price [42] |
| LDV/SOF | 22,558.97 | Korean List price [42] |
| LDV/SOF/RBV | 22,585.05 | Korean List price [42] |
| **Total drug cost for genotype 2** |  |  |
| SOF/RBV (12 week) | 19,515.46 | Korean List price [42] |
| SOF/RBV (16 week) | 26,020.61 | Korean List price [42] |

ASV, asunaprevir; DCV, daclatasvir; LDV, ledipasvir; RAV, resistant-associated variant; RBV, ribavirin; SOF, sofosbuvir; USD, United States Dollar

**Table B. Monitoring costs**

|  | **Cost ($USD)** | **Source** |
| --- | --- | --- |
| Hospital visit | 13.85 | Korean List price [42] |
| Transportation for hospital visit | 8.86 | National Health and Nutrition Examination Surveys (KNHANES) Report 2005 [43] |
| Laboratory test |  |  |
| CBC test | 7.78 | Korean List price [42] |
| LFT | 19.31 | Korean List price [42] |
| HCV RNA quantitative test | 147.33 | Korean List price [42] |
| RAV test for genotype 1b | 135.35 | Assumed† |
| **Total monitoring cost for genotype 1** |  |  |
| Non-genotype 1b | 641.21 | CBC, LFT: week 0, 4, 8, 12 |
|  |  | HCV RNA quantitative: week 0, 4, 12 |
|  |  |  |
| Genotype 1b RAV -ve | 925.97 | CBC, LFT: week 0, 4, 8, 12, 16, 20, 24 |
|  |  | HCV RNA quantitative: week 0, 4, 24 |
|  |  | RAV: prior to treatment initiation |
|  |  |  |
| Genotype 1b RAV +ve | 776.56 | CBC, LFT: week 0, 4, 8, 12 |
|  |  | HCV RNA quantitative: week 0, 4, 12 |
|  |  | RAV: prior to treatment initiation |
| **Total monitoring cost for genotype 2** |  |  |
| SOF/RBV (12 week) | 641.21 | CBC, LFT: week 0, 4, 8, 12 |
|  |  | HCV RNA quantitative: week 0, 4, 12 |
|  |  |  |
| SOF/RBV (16 week) | 691.01 | CBC, LFT: week 0, 4, 8, 12, 16 |
|  |  | HCV RNA quantitative: week 0, 4, 16 |
|  |  |  |

HCV, hepatitis C virus; CBC, complete blood count; LFT, liver function test; RAV, resistance-associated variants; RBV, rivabirin; RNA, Ribonucleic acid; SOF, sofosbuvir; USD, United States Dollar

* Based upon expert clinical opinion, it was assumed that patients visit the hospital every 4 weeks, CBC and liver function test are conducted at every visit, HCV RNA quantitative test is conducted at week 0, week 4 and at the end of treatment, and RAV test is conducted in genotype 1b prior to initiation of treatment.

† Cost for RAV test was assumed with the cost from commercial company since it has not been reimbursed yet.
